# The nucleolin MoNsr1 plays pleiotropic roles in the pathogenicity and stress adaptation in the rice blast fungus *Magnaporthe oryzae*

**DOI:** 10.3389/fpls.2024.1482934

**Published:** 2024-10-15

**Authors:** Zhen Zhang, Mohammad Shafiqul Islam, Jiuzhi Xia, Xiangyang Feng, Muhammad Noman, Jing Wang, Zhongna Hao, Haiping Qiu, Rongyao Chai, Yingying Cai, Yanli Wang, Jiaoyu Wang

**Affiliations:** ^1^ State Key Laboratory for Managing Biotic and Chemical Threats to the Quality and Safety of Agro Products, Key Laboratory of Agricultural Microbiome of MARA and Zhejiang Province, Key Laboratory of Biotechnology in Plant Protection of MARA and Zhejiang Province, Institute of Plant Protection and Microbiology, Zhejiang Academy of Agricultural Sciences, Hangzhou, China; ^2^ State Key Laboratory of Rice Biology and Breeding, Key Laboratory of Molecular Biology of Crop Pathogens and Insects of MARA, Key Laboratory of Biology of Crop Pathogens and Insects of Zhejiang Province, Institute of Biotechnology, Zhejiang University, Hangzhou, China

**Keywords:** cell wall integrity, *Magnaporthe oryzae*, MoNsr1, nucleolin, pathogenicity, stress response

## Abstract

The rice blast disease, caused by the fungus *Magnaporthe oryzae*, is a significant agricultural problem that adversely impacts rice production and food security. Understanding the precise molecular pathways involved in the interaction between the pathogen and its host is crucial for developing effective disease management strategies. This study examines the crucial function of the nucleolin MoNsr1 in regulating *M. oryzae* physiological functions. Δ*MoNsr1* deletion mutants showed reduced fungal growth, asexual sporulation, and pathogenicity compared to the wild-type. Mutants exhibited impaired conidial germination and appressoria formation, reducing infection progression. Additionally, Δ*MoNsr1* deletion mutant had less turgor pressure, confirming that MoNsr1 is essential for cell wall biogenesis and resistant to external stresses. Furthermore, Δ*MoNsr1* deletion mutant showed enhanced sensitivity to oxidative stress, reactive oxygen species, and cold tolerance. Our results offer a thorough understanding of the function of MoNsr1 in the virulence and stress-resilient capability in *M. oryzae*. These findings provide insights into the novel targets and contribute to the emergence of innovative approaches for managing rice blast disease.

## 1 Introduction

*Magnaporthe oryzae*, a disease that can reduce crop yields, is a global priority due to its economic importance as a staple meal (Dean et al., 2012; Hossain, 2022). Over the past decade, *M. oryzae* has been a key model pathogen, conducting various molecular research on fungal-plant interactions (Ebbole, 2007; Ra, 2005). So far, numerous functional genes involved in the pathogenic process of rice blast have been cloned and analyzed, which are involved in spore production, germination, appressoria formation, pathogen penetration, and post-invasion expansion (Ebbole, 2007; Galhano and Talbot, 2011; Wilson and Talbot, 2009). Several important signaling pathways involved in growth and development have been shown to affect appressorium differentiation and pathogenesis, including the G protein signaling pathway, cAMP signaling pathway, MAPK signaling pathway, and calcium ion signaling pathway etc (Li et al., 2007; Liu et al., 2022; Mehrabi et al., 2009). Up to the present, all potential genes within the rice blast fungus genome and their encoded proteins have been predicted (Li et al., 2024). This advancement facilitates comprehensive and profound research into several key fundamental life processes of the rice blast fungus.

In eukaryotic cells, the ribosomes are the factory for protein synthesis. The ribosomal synthesis starts with pre-rRNA transcription and processing, followed by ribosomal subunit assembly and nucleocytoplasmic transport (Fromont-Racine et al., 2003; Venema and Tollervey, 1999). Nucleolin is a highly abundant protein and is involved in the process of ribosomal synthesis. Nucleolin influences rDNA transcription, rRNA processing, ribosome assembly, and nucleocytoplasmic transport of ribosome particles (Cong et al., 2011; Ginisty et al., 1999; Tajrishi et al., 2011). Nucleolin usually contains three distinct structural domains: an acidic region at the N terminus and the RNA-binding domain in the middle portion. The glycine-arginine-rich (GAR) domain is found in the C-terminal region (Ginisty et al., 1999). The GAR domain plays a role in interacting with ribosomal proteins and has been reported to influence ribosomal assembly and transport (Tuteja and Tuteja, 1998).

Studying the actions of nucleolin is difficult since it performs a wide range of mechanisms that impact DNA and RNA metabolism, and it is found in various regions in subcellular locations (Mongelard and Bouvet, 2007). Nucleolin not only binds to RNA/DNA and aids in adequately folding pre-rRNA, but it also interacts with numerous proteins during the ribosome assembly process. Additionally, it functions in regulating RNA polymerase-I-based transcription (Kojima et al., 2007).

Although nucleolin was identified in the early 1980s, its function in fungi has been poorly reported. Functional studies of nucleolin have only been reported in yeast, which is primarily involved in pre-RNA processing (Gulli et al., 1995; Kondo and Inouye, 1992; Lee et al., 1992). In yeast, deletion of the nucleolin gene *ScNsr1* causes the accumulation of 35S pre-rRNA and subsequent reduction in mature 18S rRNA in the mutants and leads to the severe growth of mutant (Gulli et al., 1995; Kondo and Inouye, 1992; Lee et al., 1992), thereby *M. oryzae* MoNsr1 is likely to share a similar functions in ribosome biogenesis, involving in the processing and maturation of 35S pre-rRNA into the small subunit (18S rRNA) and the large subunit (5.8S and 25S rRNAs).

This study investigated the role of MoNsr1 homologs in *M. oryzae*. The nucleolin MoNsr1 is located in the nucleus and is essential for various processes, including vegetative growth, conidia germination, appressorium maturity, and full virulence. Additionally, the *MoNsr1* gene is crucial for maintaining cell wall integrity, and its deletion leads to increased sensitivity to reactive oxygen species (ROS) and cold.

## 2 Materials and methods

### 2.1 Fungal strains and culture conditions

In this study, the wild-type strain Guy11 of rice blast fungus was used to investigate the functions of the MoNsr1. Phenotypic analysis of vegetative growth, chemical sensitivity, and asexual sporulation of rice blast fungus mutants was performed in a complete medium (CM) (Talbot et al., 1993). After the rice blast fungus was inoculated on CM plates and grown at 28 °C with 12-hour light-dark alternation for 9 days, conidia were obtained by washing the colonies with 10 ml sterile water and filtering them through 4 layers of sterile lens paper. After the rice blast fungus was inoculated into liquid CM medium and cultured in the dark at 28°C for 3 days, the culture was collected, and genomic DNA was recovered using EZNA^®^ Fungal DNA Mini Kit (Omega Bio-tek, Inc., Norcross, GA).

### 2.2 Subcellular localization of MoNsr1

In order to investigate the subcellular localization of the MoNsr1 protein, an integrated expression plasmid for MoNsr1 and GFP was constructed using plasmid p1300BAR as the basic skeleton (Li et al., 2014). The whole *MoNsr1* gene fragment (containing the 1.5kb promoter and *MoNsr1* without a stop codon), as well as the *GFP* gene fragment, will be inserted between the *Eco*RI and *Xba*I restriction enzyme cleavage sites of plasmid p1300BAR. After the obtained plasmid *pMoNsr1-GFP* was sequenced and confirmed to be correct, the wild-type strain was introduced using the *Agrobacterium tumefaciens-*mediated transformation (*At*MT) method described by (Rho et al., 2001). Green fluorescent transformants were obtained for subcellular localization investigation of MoNsr1 using CM plate screening containing 200 μg/ml glufosinate ammonium and fluorescence microscopy (ZEISS Imager. A2). The nuclear localization of rice blast fungus was performed using the DAPI staining method (Odenbach et al., 2009), and the subcellular localization of MoNsr1 was observed and recorded under a laser confocal microscope (ZEISS LSM780).

### 2.3 Targeted gene replacement and complementation

*MoNsr1* gene-deletion strains were created according to the principle of homologous recombination. In short, the upstream and downstream fragments of the *MoNsr1* gene were first amplified using a wild-type genomic DNA template (approximately 1.0 kb each) and cloned into the plasmid p1300-KO (Li et al., 2014), respectively, between the restriction enzyme cleavage sites *Eco*RI and *Sma*I, and between *Bam*HI and *Xba*I. After obtaining the plasmid pKO-Nsr1 and confirming its correctness through sequencing, it was used for the *At*MT transformation of wild-type strains. Gene deletion mutants were obtained through CM plate screening and PCR validation containing 200 μg/ml hygromycin B. The primers used for PCR validation are shown in **Supplementary Table S1**. The *MoNsr1* gene, to obtain a complementary mutant, was amplified using the wild-type genomic DNA as a template, including the promoter region (located 1.5 kb upstream of the start codon), and cloned between the *Eco*RI and *Xba*I restriction endonuclease sites of plasmid p1300BAR. The obtained complement plasmid was introduced into a *MoNsr1* gene deletion mutant, and the complemented mutant was obtained through CM plate screening and PCR validation containing 200 μg/ml glufosinate-ammonium.

### 2.4 Pathogenicity assays

Referring to previous method (Liu et al., 2016a), pathogenicity testing was conducted on the susceptible rice (cv. CO-39) seedlings and 7-day-old barley (cv. ZJ-8) plants at 3-4 true leaf stage. The rice plants were inoculated with 0.25% gelatin suspension with a concentration of 1 × 10^5^ conidia/ml by spray. After inoculation, the rice plants were moved into a dark room with a humidity of 25 °C and above 95% for dark cultivation for 24 hours and then continued to grow alternately in light and dark for 16/8 hours at the same temperature until the wild-type inoculated plants showed typical symptoms. The disease severity of the leaves of inoculated rice plants was investigated and recorded to determine the pathogenicity of all tested strains. For the detached barley leaf inoculation assay, 20 µL of gradient diluted conidia suspension was spot inoculated on the barley leaves. The leaves were cultured in the dark at 25°C for 24 h and then illuminated until the wild-type inoculation treatment showed typical symptoms. The disease severity of each strain was compared and recorded. The experiment was carried out at least three times, with three replicates each time, to obtain similar results.

### 2.5 Analysis of infection-related morphogenesis

20 μL of 5 × 10^4^ conidia/ml suspension was inoculated onto a plastic coverslip (Thermo Fisher Scientific, Waltham, MA, USA) and cultured in the dark at 25°C. Conidia germination and appressorium differentiation were observed and recorded under a microscope for 2h, 4h, 8h, 12h and 24h after inoculation. The appressorium turgor pressure of rice blast fungus was determined according to the previous method (Dixon et al., 1999). The appressorium formed by induction on the hydrophobic surface for 24h was treated with 1-4 M glycerol for 10 minutes, and the collapse rate of mutant and wild-type appressorium after glycerol treatment was counted under a microscope. The test was repeated three times, and the number of conidia or appressorium observed for each strain was no less than 200. For the rice blast infection rate test, barley leaves grown for 7 days were selected and sprayed with 1 × 10^-5/^mL rice blast conidia suspension. After 36 h or 48 h of inoculation, the barley leaves were decolorized with methanol, and the number of infected appressorium was counted under a light microscope. The experiment was repeated 3 times, and no less than 50 appressorium was observed for each strain.

### 2.6 Phosphorylation assays

The mycelium of all tested strains grown in liquid CM was used to extract the proteins. Western blotting was used to identify the amounts of phosphorylated Pmk1 and Mps1. To detect phosphor-Mps1 and phospho-Pmk1, a primary anti-phospho-p44/42 MAPK antibody (Cell Signaling Technology, USA) was employed. Two antibodies were applied to detect Mps1 and Pmk1, anti-ERK1/2 MAPK antibody (Cell Signaling Technology, USA) for Mps1 and anti-p44/42 MAPK antibody (Santa Cruz, USA) for Pmk1. A monoclonal anti-GAPDH antibody (HUABIO, China) was also used to detect the samples. Using the ImageJ program, the band intensity was calculated.

### 2.7 Statistical analysis

The relevant experimental data were processed with Excel 2016, and one-way ANOVA was analyzed using DPS (Data Processing System) v20.05. Significance levels were determined using Duncan’s new multiple-range test at *p* < 0.05.

## 3 Results

### 3.1 Identification of MoNsr1

**Figure 1:**
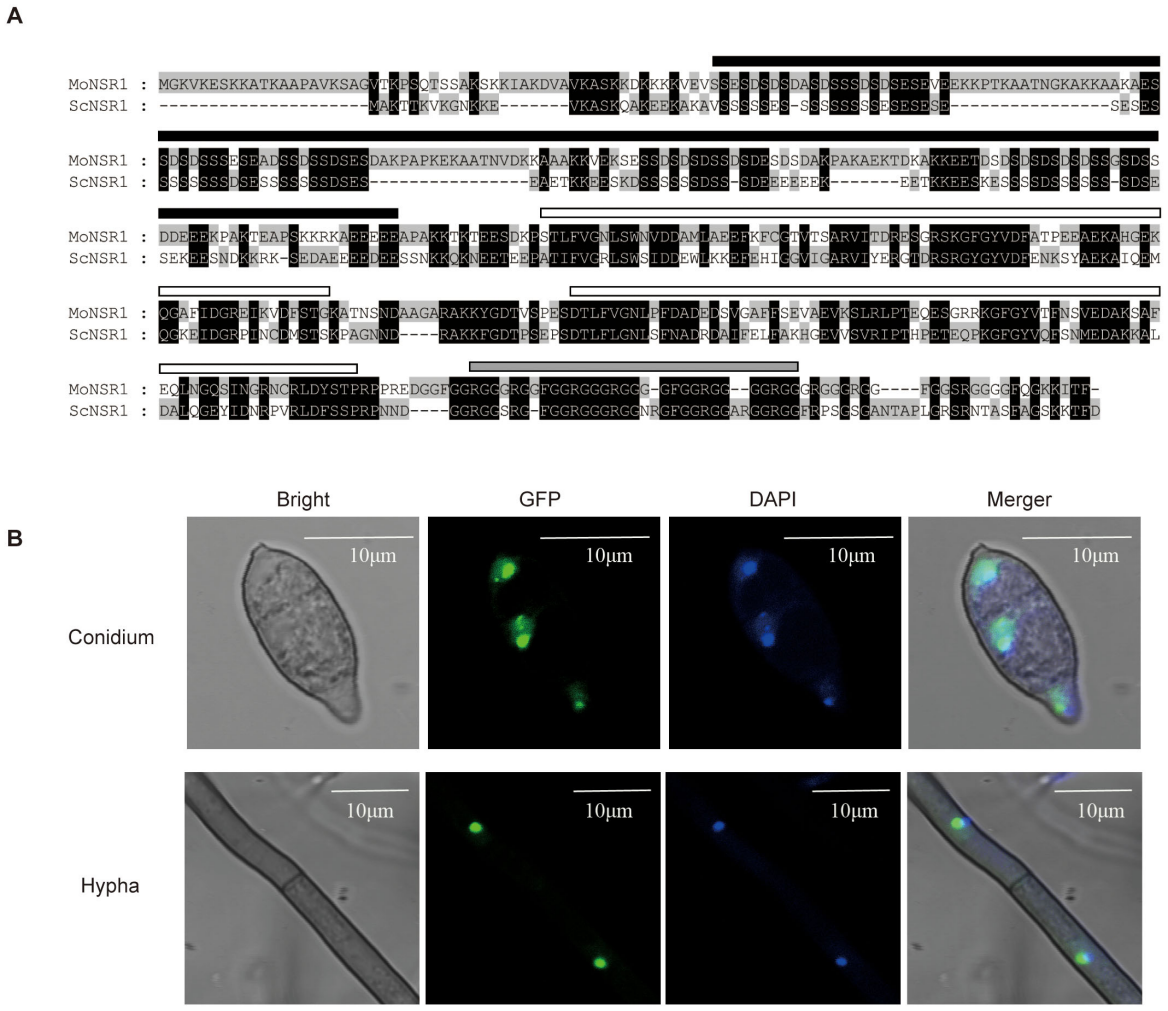
Primary structure and subcellular localization of MoNsr1. **(A)** Comparison of amino acid sequence of MoNsr1 and ScNsr1. The conserved amino acids are black-shaded. The black rectangle represents the acidic N-terminal domain, the white rectangles show the two RRM domains and the gray rectangle represents the GAR domain. **(B)** The MoNsr1-GFP protein was co-localized with DAPI-stained DNA in *M. oryzae*. Bar = 10 μm.

The homologous genes of *Saccharomyces cerevisiae* ScNsr1 were searched in the EsdmblFungi (https://fungi.ensembl.org) database using the BLASTp program. The resultant MoNsr1 (MGG_01268) was obtained and comprised 486 amino acids. The MoNsr1-encoding gene comprises 1592 bp, with 2 introns and an exon. After amino acid sequence alignment, MoNsr1 showed 54% homology with ScNsr1 (**Figure 1A**), including 4 conserved domains. The N-terminus includes an acidic domain composed of serine and acidic residues. This structure may be related to the interaction between nucleolin and ribosomal proteins, histone H1, U3 snoRNP, etc (Erard et al., 1988; Ginisty et al., 1998; Sicard et al., 1998). The middle part contains two RRMs (RNA recognition motifs), which allow the protein to bind to RNA specifically, and the C-terminal GAR domain. The GAR domain is often found in nucleolar proteins, and the GAR domain in nucleolin interacts with ribosomal proteins (Bouvet et al., 1998).

### 3.2 MoNsr1 is localized to the nucleus

In yeast, *Nsr*1 is localized in the nucleus and binds to the (TG1-3)_n_ telomeric DNA (Lee et al., 1991; Lin and Zakian, 1994). The MoNsr1-GFP fusion expression vector constructed in this study was transformed into the rice blast fungus strain Guy11 by *At*MT, and 6 transformants with detectable green fluorescence were obtained. There was no significant difference in the obtained transformants growth and sporulation phenotypes compared with the wild-type. The hyphae and spores of the GFP-labeled transformants were stained with 0.8 μg/ml DAPI solution and observed under a fluorescence microscope. The blue nuclei labeled with DAPI fluorescence in the cells co-localized with the green fluorescence of the MoNsr1-GFP fusion expression protein (**Figure 1B**). The results showed that MoNsr1, like ScNsr1, is localized in the nucleus.

### 3.3 Deletion of *MoNsr1* affected the vegetative growth and asexual reproduction in *M. oryzae*

**Figure 2:**
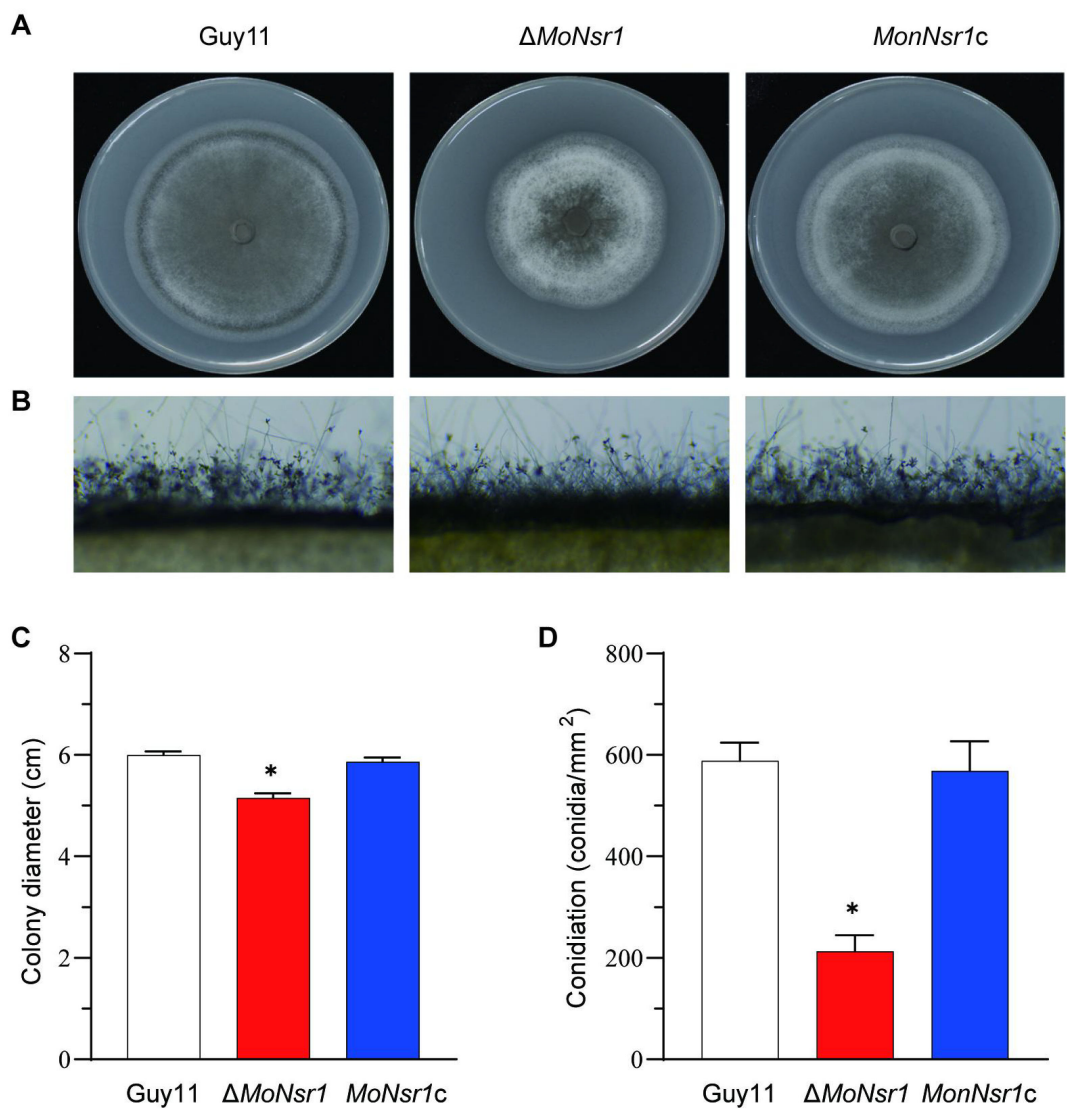
Vegetative growth and conidiation of *M. oryzae* strains in CM medium. **(A)** Colony morphology on CM plates for 6 days at 28°C with 12 h light and dark alternation. **(B)** Development of conidia on conidiophores on CM. **(C)** Statistical analysis of the vegetative growth rate of the tested strain on CM plates. **(D)** Statistical analysis of the number of conidia on CM plates. Asterisks in each data column indicate significant differences at *p* < 0.05.

We performed gene knockout and complementation to investigate the function of the *MoNsr1* gene. Inoculate the wild-type strain, gene deletion mutant Δ*MoNsr1*, and the complementary strain *MoNsr1*c onto CM medium with alternate light and dark cultivation at 28 °C for 12 hours for 6 days. The results showed that the average colony diameter of the Δ*MoNsr1* mutant was significantly smaller than that of the wild-type (p<0.05) (**Figures 2A, C**). Rectangular mycelium blocks grown on CM medium for 4 days were cut and placed on a glass slide. The conidia induction experiment was carried out by alternating light and dark at 28 °C for 12 and 48 hours. Microscopic observation confirmed that compared with the wild-type and complemented strains, the Δ*MoNsr1* mutant had fewer conidiophores and produced sparse conidia (**Figure 2B**). Conidia grown on CM medium for 6 days were collected for statistics. The results showed that the yield of conidia per unit area of the Δ*MoNsr1* mutant was only 35% of that of the wild-type (**Figure 2D**). The above results indicate that the loss of the *MoNsr1* gene is involved in the vegetative growth and asexual reproduction of rice blast fungus.

### 3.4 MoNsr1 is indispensable for full virulence of *M. oryzae*

**Figure 3:**
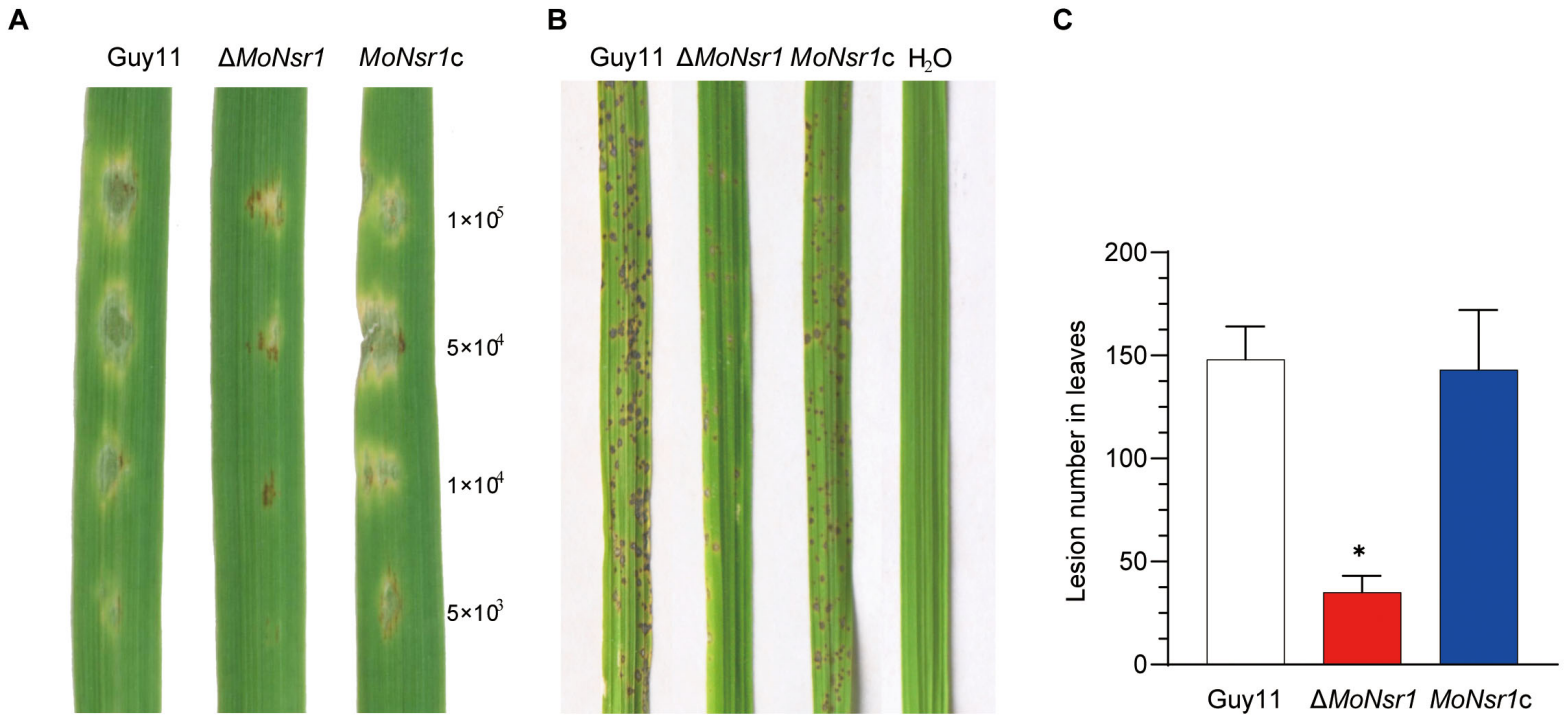
Pathogenicity of the tested *M. oryzae* strains. **(A)** Drop inoculation with 20 μL serial dilutions of conidia suspension on barley leaf segments. **(B)** Spray inoculation with 14-day-old rice seedlings. Representative leaves were photographed 6 days post-inoculation. **(C)** Lesion number in rice leaves. Asterisks in each data column indicate significant differences at *p* < 0.05.

In order to clarify the role of the MoNsr1 in the pathogenesis of rice blast fungus, the wild-type strain Guy11, gene deletion mutant Δ*MoNsr1*, and compensatory strain *MoNsr1*c were used to inoculate barley leaves and 14-day-old susceptible rice seedlings (CO-39). The pathogenicity results of detached barley leaves showed that the pathogenicity of the Δ*MoNsr1* mutant was lower than that of wild-type and complement treated with the same concentration of spore solution. Pathogenicity weakening became more pronounced with decreased spore concentration (**Figure 3A**). The results of inoculation of rice seedlings with 1 x 10^5^ spores/ml by spray showed that the mutant Δ*MoNsr1* produced fewer disease spots, and the disease severity significantly decreased compared with that of the wild-type strain (**Figures 3B, C**).

### 3.5 MoNsr1 is involved in the germination of conidia and maturation of appressorium

**Figure 4:**
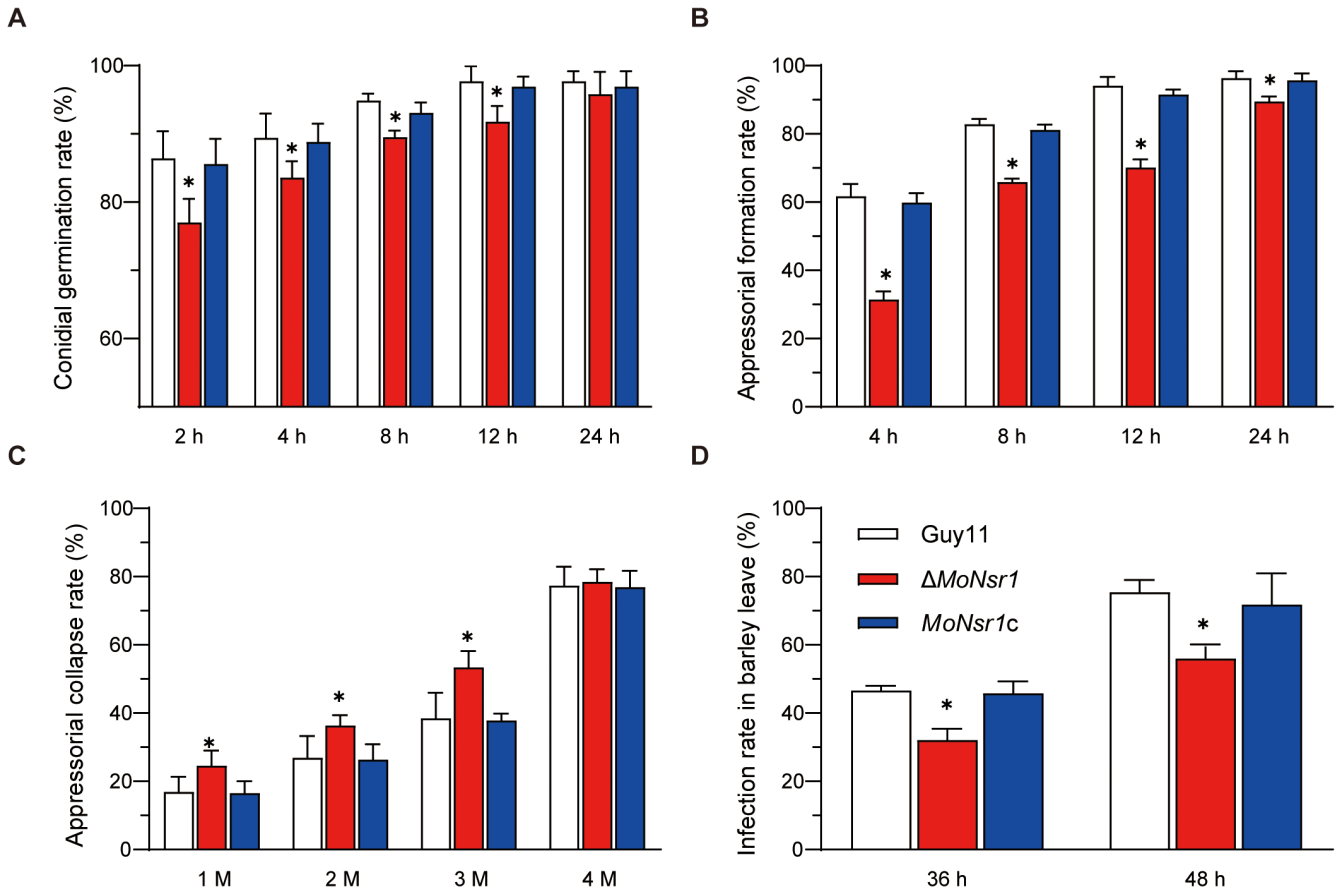
Analysis of infection-related morphogenesis of Δ*MoNsr1* deletion mutant. **(A)** conidial germination rate; **(B)** appressorial formation rate; **(C)** appressorial collapse rate; **(D)** Infection rate in barley leaf cells. Asterisks in each data column indicate significant differences at *p* < 0.05.

In order to investigate the impact of *MoNsr1* gene deletion on the pathogenicity of rice blast fungus, we analyzed the effects of gene deletion mutants on conidia germination, appressorium differentiation, and host epidermal penetration ability. The results of the conidial germination experiment showed that the conidia of the Δ*MoNsr1* mutant could germinate and form an appressorium. Compared with the wild-type, the conidial germination rate and appressorium formation rate of the Δ*MoNsr1* mutant were significantly reduced (**Figures 4A, B**). When the appressorium induced for 24 hours was treated with 1-4 M glycerol, the collapse rate of the appressorium in the Δ*MoNsr1* mutant was significantly higher than that in the wild-type (**Figure 4C**). These results indicated that *MoNsr1* is involved in conidial germination, appressorium differentiation, and maturation.

We analyzed the infection rate in barley leaves to determine whether the decrease in turgor pressure of the Δ*MoNsr1* mutant caused the decrease in host pathogenicity. The results showed that after inoculating barley leaves with a suspension of 5 × 10^4^ spores/ml *in vitro* for 36 and 48 hours, the infection rates of the Δ*MoNsr1* mutant on barley epidermal cells decreased by 31% and 26%, respectively, compared to that of the wild-type under a microscope (**Figure 4D**). The above indicates that the absence of the MoNsr1 leads to a decrease in mutant infection rate, resulting in a decrease in mutant pathogenicity. This result is consistent with the decrease in appressorial turgor pressure in the Δ*MoNsr1* mutant.

### 3.6 MoNsr1 is involved in cell wall integrity

**Figure 5:**
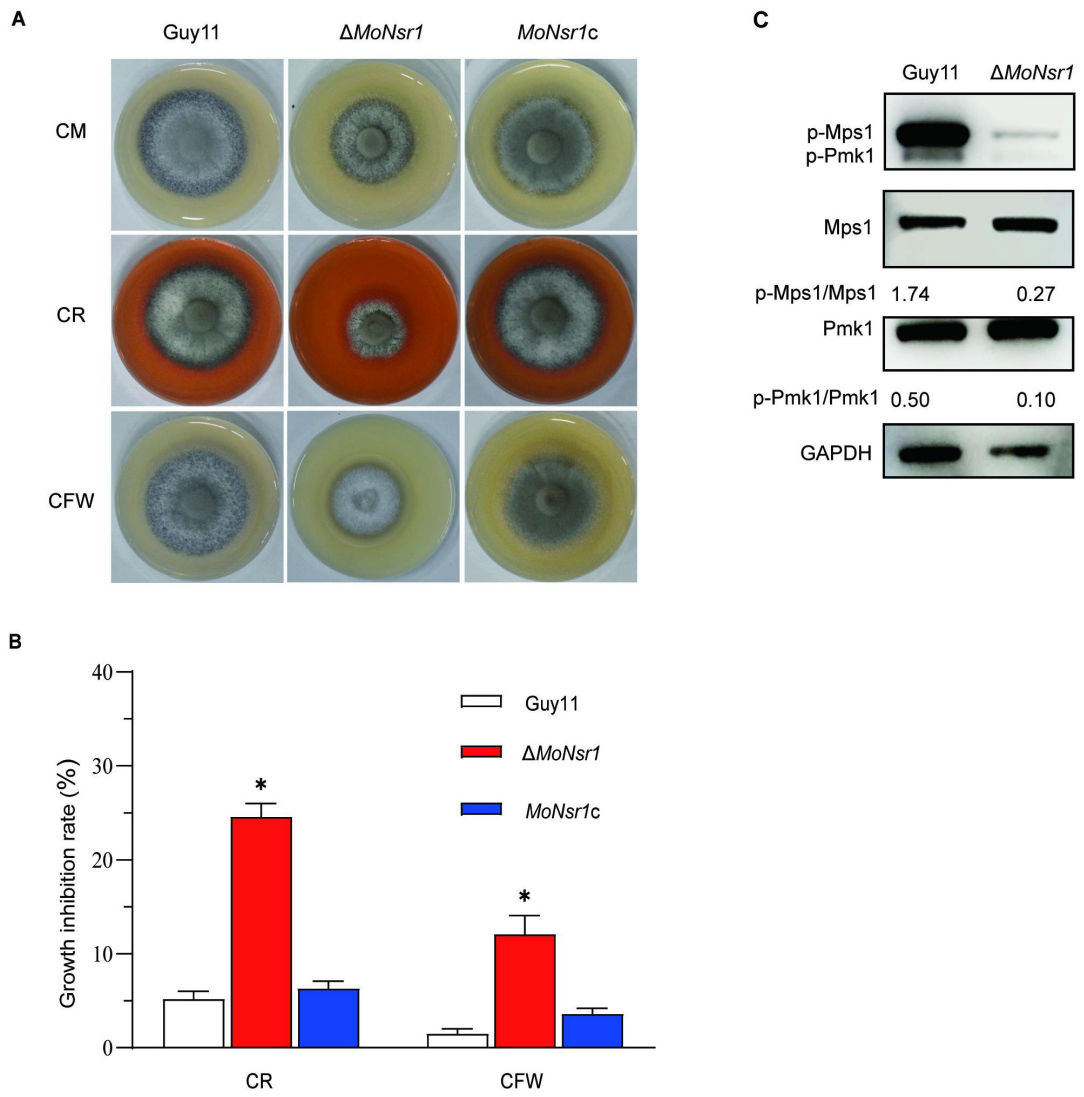
Effect of cell wall disruptors on vegetative growth of the tested strains. **(A)** Colony morphology of the tested strains grown on CM containing Congo red (25 μg/mL) or Calcolflour white (12.5 μg/mL) at 28°C for 6 days; **(B)** Relative inhibition rate of the tested strains under Congo red or Calcolflour White stress; **(C)** The phosphorylation level of Mps1 and Pmk1 in the tested strains. Asterisks in each data column indicate significant differences at *p* < 0.05.

We investigated the mechanism effects of the MoNsr1 on the cell wall integrity of the gene deletion mutant and the sensitivity of the Δ*MoNsr1* mutant to cell wall disruptors. The results showed that the growth of the Δ*MoNsr1* mutant was inhibited on CM plates containing 25 μg/mL Congo red (CR) and 12.5 μg/mL Calcolflour white (CFW), with inhibition rates of 24.6% and 12.1%, respectively, which were significantly higher than those of the wild-type (**Figures 5A, B**). The results indicate that gene deletion leads to defects in the integrity of the Δ*MoNsr1* mutant cell wall. The cell wall integrity signaling pathway is a key mechanism for sensing and adapting to various environmental conditions, and the Mps1-mediated MAPK signaling pathway is its core component (Lu et al., 2023). The phosphorylation level of Mps1 in the Δ*MoNsr1* mutant was analyzed, and the results showed that compared with the wild-type, the Mpsl phosphorylation level of the Δ*MoNsr1* mutant was lower (**Figure 5C**). The above results indicate that MoNsr1 may respond to the cell integrity signaling pathway by regulating Mpsl phosphorylation. Surprisingly, the phosphorylation level of Pmkl in the Δ*MoNsr1* mutant was also lower than that of the wild-type. Pmk1 is an important signaling pathway that regulates appressorium differentiation and infection in rice blast fungus (Zhang et al., 2011). Therefore, MoNsr1 may also participate in the differentiation of appressorium by regulating the phosphorylation of Pmk1.

### 3.7 *MoNsr1* deletion results in sensitivity to ROS and cold

**Figure 6:**
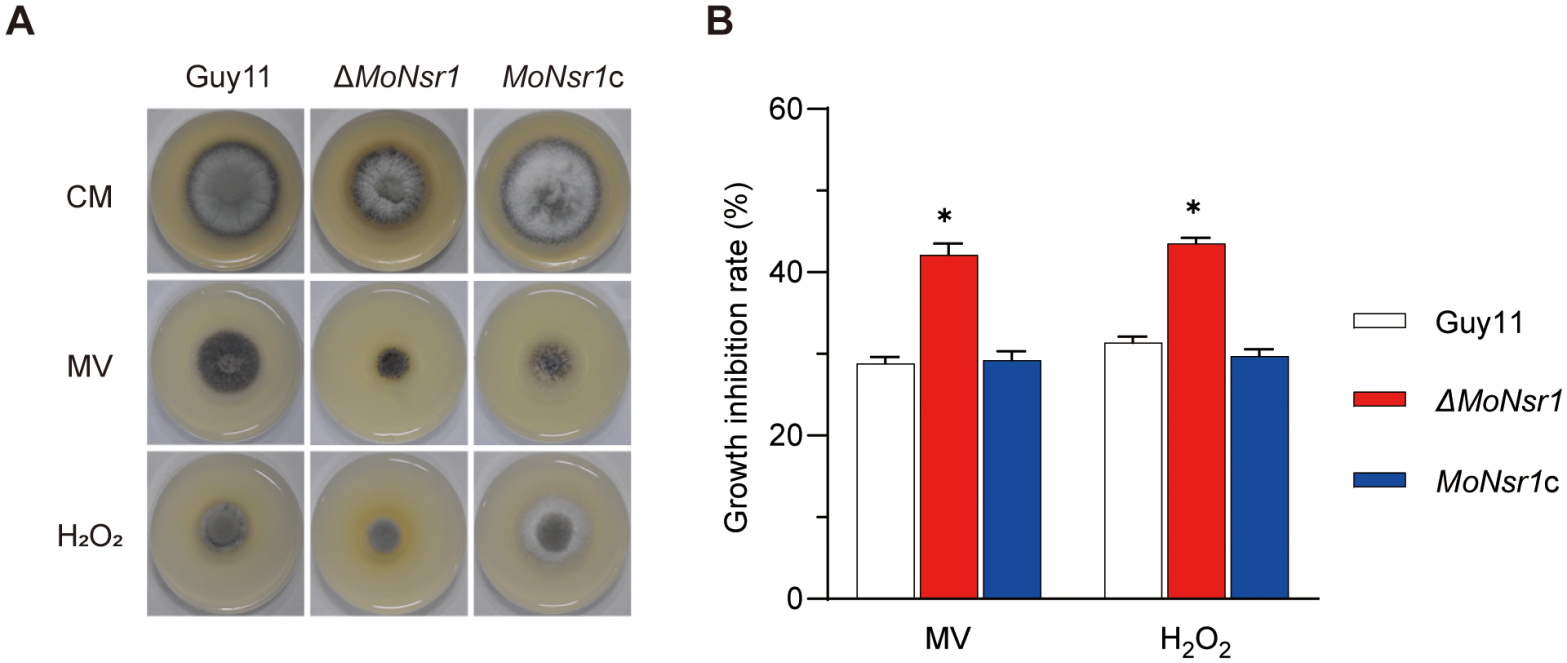
Effect of oxidants on vegetative growth of the tested *M. oryzae* strains. **(A)** Colony morphology of the tested strains grown on CM containing methyl viologen (MV, 2.0 mM) and H_2_O_2_ (7.35 mM) at 28°C for 6 days; **(B)** Relative inhibition rate under ROS stress. Asterisks in each data column indicate significant differences at *p* < 0.05.

**Figure 7:**
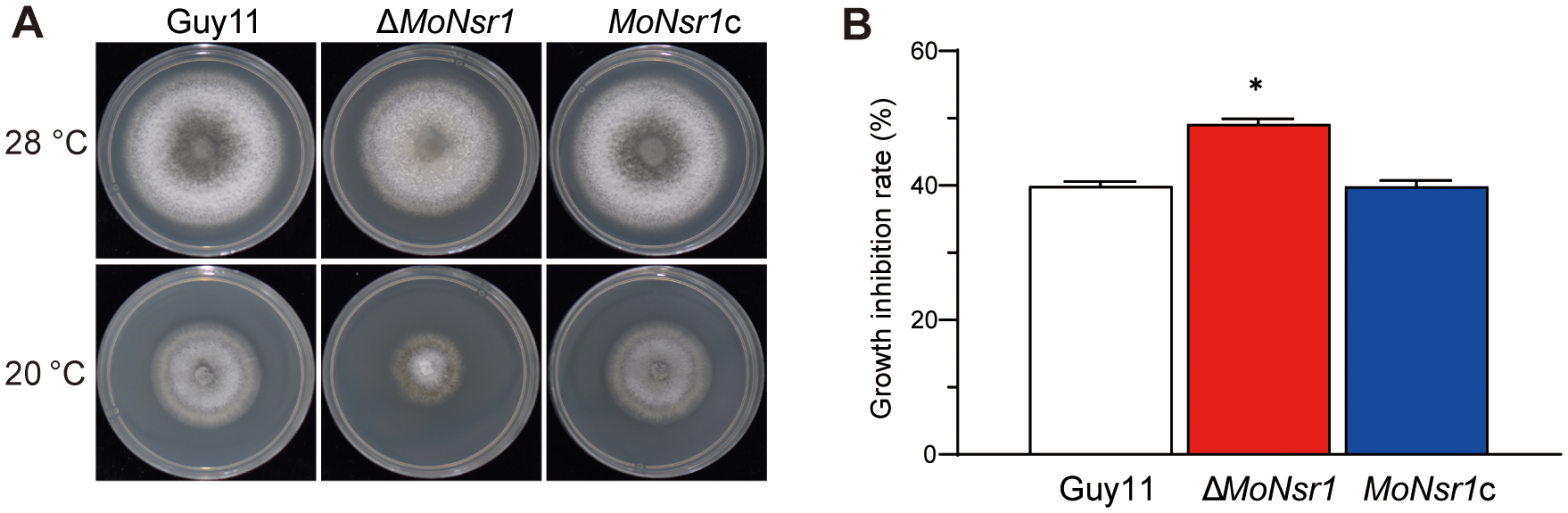
Effect of cold on vegetative growth of the tested *M. oryzae* strains. **(A)** Colony morphology of the tested strains grown on CM at 28°C and 20°C for 6 days; **(B)** Relative inhibition rate under cold stress. Asterisks in each data column indicate significant differences at *p* < 0.05.

In addition, we also found that the Δ*MoNsr1* mutant is more sensitive to oxidants and cold stress. The relative growth inhibition rates of mutants on CM plates containing 2 mM methyl viologen (MV) or 7.35 mM H_2_O_2_ were 42% and 43%, respectively, significantly lower than that of the wild-type (**Figures 6A, B**). The relative growth inhibition rate of mutants cultured at 20 °C was 49%, significantly higher than that of the wild-type (40%) (**Figures 7A, B**). These findings indicate that MoNsr1 plays a positive role in resisting oxidative and low-temperature stress.

## 4 Discussion

Nucleolin is a multifunctional non-ribosomal protein, which directly interacts with the pre-rRNA by its RRM domains to participate in the cleavages of pre-rRNA A1 and A2 and interact with ribosomal proteins to participate in pre-ribosomal particle assembly (Ginisty et al., 1998). Several potential CK2 phosphorylation sites were found in the N-terminal acidic region. The phosphorylated nucleolin regulates cell growth, proliferation, apoptosis and DNA damage response (Xiao et al., 2014; Zhang et al., 2018). In the present study, a yeast nucleolin ScNsr1 homolog in *M. oryzae*, MoNsr1, was identified. MoNsr1 has a similar protein structure to ScNsr1 and is located in the nucleus. The homology in nuclear distribution and protein structure between MoNsr1 and ScNsr1 implies that both proteins likely have comparable functions, such as being involved with ribosomal RNA processing, as evidenced by the presence of RRM and GAR domains in their sequences. Due to defects of growth, spore production, and pathogenicity of gene deletion mutants, it was implied that nucleolin MoNsr1 plays a pivotal role in crucial cellular processes required for growth.

The identification of MoNsr1 functions in the processing of ribosomal RNA and its influence on cellular activities including growth and proliferation improves comprehension of the complex molecular mechanisms present in *M. oryzae.* Ribosomes in eukaryotic cells are responsible for protein translation, which biosynthesis is a complex process involving the coordination of rDNA, rRNA, RNA polymerase I, and a lot of the non-ribosomal or ribosomal proteins. Nucleolin is involved in pre-rRNA cleavage to produce mature 18S rRNA (Gulli et al., 1995). The loss function of nucleolin affects the accumulation of 40S ribosomal subunits. In *M. oryzae*, it was documented that ribosome biosynthesis plays an important role in growth and pathogenicity (Cao et al., 2016; Li et al., 2019; Liu et al., 2016b, 2018). For example, ScYvh1 and ScRei1 are involved in 60S ribosomal subunit biogenesis (Meyer et al., 2010), and ScFap7 is a ribosome assembly factor involved in 40S ribosomal subunit biogenesis (Kemmler et al., 2009).

The phenotype of Δ*MoNsr1* mutants in vegetative growth, asexual reproduction, pathogenicity, cell wall, and oxidative stress were similar to that of the mutants of the above three genes. Given that nucleolin plays an early role in ribosome synthesis, the evidence suggests that MoNsr1 might participate in the early stage of ribosomal biosynthesis. This participation could subsequently influence the development and pathogenicity of the rice blast fungus, making further studies focusing on this hypothesis essential for understanding its impact on crop health. The correlation between MoNsr1 and ribosome biosynthesis has significant effects on concentrating on crucial individuals involved in the life cycle of the pathogen, like MoNsr1, we can interrupt the progression of the disease and promote the development of robust crops.

Mitogen-activated protein kinase (MAPK) cascade responds to host and environmental signals in *M. oryzae* (Mehrabi et al., 2009). The Mps1-mediated MAPK signaling pathway regulates penetration, conidiation, and cell wall integrity in *M. oryzae*. The Δ*Mps1* mutant is reduced in aerial hyphal growth and conidiation and is hypersensitive to cell wall disruptors (Xu et al., 1998). The suppression of the Δ*MoNsr1* mutant growth on media containing CR and CFW provides evidence to support the concept that *MoNsr1* plays a role in maintaining the cell wall. This data, supported by additional evidence, shows a reduction in the phosphorylation level of Mps1 in the Δ*MoNsr1*. Significantly, they also discovered a decreased degree of phosphorylation of Pmk1 in the Δ*MoNsr1* mutant, which is a crucial regulator of appressorium differentiation and infection (Xu and Hamer, 1996; Sakulkoo et al., 2018; Zhang et al., 2011). The complex regulatory network that controls the pathogen’s interaction with the host is indicated by the influence of MoNsr1 on MAPK signaling pathways and the association with cell wall integrity and stress responses. Develop measures that can lessen the adverse impacts of *M. oryzae* and other associated diseases on crop health, these pathways must be understood.

Interestingly, we found that the mutants were susceptible to cold stress. The cold stress response in yeast is transduced by the Hog1-mediated MAPK signaling pathway (Panadero et al., 2006). These findings indicate that *MoNsr*1 might have a broader function in the infection process and cold stress, potentially playing a role in MAPK signaling pathways. The increased vulnerability of *MoNsr1*-deficient mutants to cold stress emphasizes the protein’s wider involvement in *M. oryzae* ability to adjust to different environmental circumstances.

Based on the growth of the Δ*MoNsr1* mutant on the medium containing H_2_O_2_, it was speculated that the growth of the infected hyphae of the mutant could be blocked within the host, indicating a potential limitation in its pathogenicity. Previous investigations have demonstrated that MoYvh1 can increase ribosome production, which promotes the synthesis and secretion of ROS-scavenging extracellular proteins, allowing the infected mycelium to grow in host tissue under oxidative stress (Liu et al., 2018). Thus, we speculate that ribosome biosynthesis in the Δ*MoNsr1* mutant may also be affected, leading to removing the ROS extracellular protein synthesis and increasing the sensitivity to ROS.

Our results revealed that nucleolin *MoNsr1* plays a crucial significance in the biology of this destructive rice blast. MoNsr1 is a multifunctional protein required for vegetative growth, conidiogenesis, pathogenicity, and the MAPK pathways. Discovering the various activities of MoNsr1 offers potential for future research, promoting greater exploration of the complex interplay between fungal genes and their impact on disease-causing capability and environmental adaption.

## 5 Conclusions

The results of this study provide significant insights into the role of the nucleolin gene, *MoNsr1*, in the pathogenicity and stress adaptation of the rice blast fungus, *M. oryzae*. The findings demonstrate that MoNsr1 is crucial for various physiological processes, including vegetative growth, asexual reproduction, and full virulence of the pathogen. The deletion of *MoNsr1* led to reduced fungal growth, impaired conidial germination, and defective appressorium formation, all of which are critical factors in the infection process. Furthermore, the Δ*MoNsr1* deletion mutant exhibited decreased turgor pressure, confirming the essential role of MoNsr1 in maintaining cell wall integrity. The increased sensitivity to oxidative stress and cold tolerance in the mutants highlights the importance of MoNsr1 in stress response mechanisms. Overall, these findings underline the pivotal function of MoNsr1 in the virulence and stress adaptability of *M. oryzae*, offering potential novel targets for developing strategies to control rice blast disease.

## Data Availability

The original contributions presented in the study are included in the article/**Supplementary Material**. Further inquiries can be directed to the corresponding author.

## References

[B1] Bouvet P. Diaz J. J. Kindbeiter K. Madjar J. J. Amalric F. (1998). Nucleolin interacts with several ribosomal proteins through its RGG domain. J. Biol. Chem. 273, 19025–19029. doi: 10.1074/jbc.273.30.19025 9668083

[B2] Cao H. Huang P. Zhang L. Shi Y. Sun D. Yan Y.. (2016). Characterization of 47 Cys_2_-His_2_ zinc finger proteins required for the development and pathogenicity of the rice blast fungus *Magnaporthe oryzae* . New Phytol. 211, 1035–1051. doi: 10.1111/nph.2016.211.issue-3 27041000

[B3] Cong R. Das S. Bouvet P. (2011). “The multiple properties and functions of nucleolin,” in The Nucleolus (Springer New York, New York, NY), 185–212.

[B4] Dean R. Van Kan J. A. L. Pretorius Z. A. Hammond-Kosack K. E. Di Pietro A. Spanu P. D.. (2012). The Top 10 fungal pathogens in molecular plant pathology. Mol. Plant Pathol. 13, 414–430. doi: 10.1111/j.1364-3703.2011.00783.x 22471698 PMC6638784

[B5] Dixon K. P. Xu J. R. Smirnoff N. Talbot N. J. (1999). Independent signaling pathways regulate cellular turgor during hyperosmotic stress and appressorium-mediated plant infection by *Magnaporthe grisea* . Plant Cell 11, 2045–2058. doi: 10.1105/tpc.11.10.2045 10521531 PMC144108

[B6] Ebbole D. J. (2007). *Magnaporthe* as a model for understanding host-pathogen interactions. Annu. Rev. Phytopathol. 45, 437–456. doi: 10.1146/annurev.phyto.45.062806.094346 17489691

[B7] Erard M. S. Belenguer P. Caizergues-Ferrer M. Pantaloni A. Amalric F. (1988). A major nucleolar protein, nucleolin, induces chromatin decondensation by binding to histone Hl. Eur. J. Biochem. 175, 525–530. doi: 10.1111/j.1432-1033.1988.tb14224.x 3409881

[B8] Fromont-Racine M. Senger B. Saveanu C. Fasiolo F. (2003). Ribosome assembly in eukaryotes. Gene 313, 17–42. doi: 10.1016/S0378-1119(03)00629-2 12957375

[B9] Galhano R. Talbot N. J. (2011). The biology of blast: Understanding how *Magnaporthe oryzae* invades rice plants. Fungal Biol. Rev. 25, 61–67. doi: 10.1016/j.fbr.2011.01.006

[B10] Ginisty H. Amalric F. Bouvet P. (1998). Nucleolin functions in the first step of ribosomal RNA processing. EMBO J. 17, 1476–1486. doi: 10.1093/emboj/17.5.1476 9482744 PMC1170495

[B11] Ginisty H. Sicard H. Roger B. Bouvet P. (1999). Structure and functions of nucleolin. J. Cell Sci. 112, 761–772. doi: 10.1242/jcs.112.6.761 10036227

[B12] Gulli M. P. Girard J. P. Zabetakis D. Lapeyre B. Melese T. Caizergues-Ferrer M. (1995). Gar2 is a nucleolar protein from *Schizosaccharomyces pombe* required for 18S rRNA and 40S ribosomal subunit accumulation. Nucleic Acids Res. 23, 1912–1918. doi: 10.1093/nar/23.11.1912 7596817 PMC306962

[B13] Hossain M. M. (2022). Wheat blast: A review from a genetic and genomic perspective. Front. Microbiol. 13, 983243. doi: 10.3389/fmicb.2022.983243 36160203 PMC9493272

[B14] Kemmler S. Occhipinti L. Veisu M. Panse V. G. (2009). Yvh1 is required for a late maturation step in the 60S biogenesis pathway. J. Cell Biol. 186, 863–880. doi: 10.1083/jcb.200904111 19797079 PMC2753168

[B15] Kojima H. Suzuki T. Kato T. Enomoto K. Sato S. Kato T.. (2007). Sugar-inducible expression of the nucleolin-1 gene of *Arabidopsis thaliana* and its role in ribosome synthesis, growth and development. Plant J. 49, 1053–1063. doi: 10.1111/j.1365-313X.2006.03016.x 17286797

[B16] Kondo K. Inouye M. (1992). Yeast NSR1 protein that has structural similarity to mammalian nucleolin is involved in pre-rRNA processing. J. Biol. Chem. 267, 16252–16258. doi: 10.1016/S0021-9258(18)41993-X 1644811

[B17] Lee W. C. Xue Z. Mélèse T. (1991). The NSR1 gene encodes a protein that specifically binds nuclear localization sequences and has two RNA recognition motifs. J. Cell Biol. 113, 1–12. doi: 10.1083/jcb.113.1.1 1706724 PMC2288927

[B18] Lee W. C. Zabetakis D. Mélèse T. (1992). NSR1 is required for pre-rRNA processing and for the proper maintenance of steady-state levels of ribosomal subunits. Mol. Cell. Biol. 12, 3865–3871. doi: 10.1128/mcb.12.9.3865-3871.1992 1508189 PMC360260

[B19] Li L. Wang J. Zhang Z. Wang Y. Liu M. Jiang H.. (2014). MoPex19, which is essential for maintenance of peroxisomal structure and woronin bodies, is required for metabolism and development in the rice blast fungus. PLoS One 9, e85252. doi: 10.1371/journal.pone.0085252 24454828 PMC3891873

[B20] Li L. Wright S. J. Krystofova S. Park G. Borkovich K. A. (2007). Heterotrimeric G protein signaling in filamentous fungi. Annu. Rev. Microbiol. 61, 423–452. doi: 10.1146/annurev.micro.61.080706.093432 17506673

[B21] Li L. Zhu X. M. Shi H. B. Feng X. X. Liu X. H. Lin F. C. (2019). MoFap7, a ribosome assembly factor, is required for fungal development and plant colonization of *Magnaporthe oryzae* . Virulence 10, 1047–1063. doi: 10.1080/21505594.2019.1697123 31814506 PMC6930019

[B22] Li Z. Yang J. Ji X. Liu J. Yin C. Bhadauria V.. (2024). First telomere-to-telomere gapless assembly of the rice blast fungus *Pyricularia oryzae* . Sci. Data 11, 380. doi: 10.1038/s41597-024-03209-z 38615081 PMC11016069

[B23] Lin J. J. Zakian V. A. (1994). Isolation and characterization of two *Saccharomyces cerevisiae* genes that encode proteins that bind to (TG_1–3_) n single strand telomeric DNA in *vitro* . Nucleic Acids Res. 22, 4906–4913. doi: 10.1093/nar/22.23.4906 7800479 PMC523755

[B24] Liu C. Liu T. Lv Z. Qin M. Qu Z. Zhang Z.. (2022). A calcineurin regulator MoRCN1 Is Important for asexual development, stress response, and plant infection of *Magnaporthe oryzae* . Front. Plant Sci. 13, 925645. doi: 10.3389/fpls.2022.925645 35783935 PMC9244802

[B25] Liu X. Cai Y. Zhang X. Zhang H. Zheng X. Zhang Z. (2016a). Carbamoyl phosphate synthetase subunit MoCpa2 affects development and pathogenicity by modulating arginine biosynthesis in *Magnaporthe oryzae* . Front. Microbiol. 7, 2023. doi: 10.3389/fmicb.2016.02023 28066349 PMC5166579

[B26] Liu X. Qian B. Gao C. Huang S. Cai Y. Zhang H.. (2016b). The putative protein phosphatase MoYvh1 functions upstream of MoPdeH to regulate the development and pathogenicity in *Magnaporthe oryzae* . Mol. Plant Microbe Interact. 29, 496–507. doi: 10.1094/MPMI-11-15-0259-R 27110741

[B27] Liu X. Yang J. Qian B. Cai Y. Zou X. Zhang H.. (2018). MoYvh1 subverts rice defense through functions of ribosomal protein MoMrt4 in *Magnaporthe oryzae* . PLoS Pathog. 14, e1007016. doi: 10.1371/journal.ppat.1007016 29684060 PMC5933821

[B28] Lu K. Chen R. Yang Y. Xu H. Jiang J. Li L. (2023). Involvement of the cell wall–integrity pathway in signal recognition, cell-wall biosynthesis, and virulence in *Magnaporthe oryzae* . Mol. Plant Microbe Interact. 36, 608–622. doi: 10.1094/MPMI-11-22-0231-CR 37140471

[B29] Mehrabi R. Zhao X. Kim Y. Xu J. R. (2009). “The cAMP signaling and map kinase pathways in plant pathogenic fungi,” in Plant Relationships (Springer Berlin Heidelberg, Berlin, Heidelberg), 157–172.

[B30] Meyer A. E. Hoover L. A. Craig E. A. (2010). The Cytosolic J-protein, Jjj1, and Rei1 function in the removal of the Pre-60 S subunit factor Arx1. J. Biol. Chem. 285, 961–968. doi: 10.1074/jbc.M109.038349 19901025 PMC2801297

[B31] Mongelard F. Bouvet P. (2007). Nucleolin: A multifaceted protein. Trends. Cell Biol. 17, 80–86. doi: 10.1016/j.tcb.2006.11.010 17157503

[B32] Odenbach D. Thines E. Anke H. Foster A. J. (2009). The *Magnaporthe grisea* class VII chitin synthase is required for normal appressorial development and function. Mol. Plant Pathol. 10, 81–94. doi: 10.1111/j.1364-3703.2008.00515.x 19161355 PMC6640330

[B33] Panadero J. Pallotti C. Rodríguez-Vargas S. Randez-Gil F. Prieto J. A. (2006). A downshift in temperature activates the high osmolarity glycerol (HOG) pathway, which determines freeze tolerance in *Saccharomyces cerevisiae* . J. Biol. Chem. 281, 4638–4645. doi: 10.1074/jbc.M512736200 16371351

[B34] Ra D. (2005). The genome sequence of the rice blast fungus *Magnaporthe grisea* . Nature 434, 980–986. doi: 10.1038/nature03449 15846337

[B35] Rho H.-S. Kang S. Lee Y. H. (2001). *Agrobacterium tumefaciens*-mediated transformation of the plant pathogenic fungus, *Magnaporthe grisea* . Mol. Cell 12, 407–411. doi: 10.1016/S1016-8478(23)17116-0 11804343

[B36] Sakulkoo W. Osés-Ruiz M. Oliveira Garcia E. Soanes D. M. Littlejohn G. R. Hacker C.. (2018). A single fungal MAP kinase controls plant cell-to-cell invasion by the rice blast fungus. Science 359, 1399–1403. doi: 10.1126/science.aaq0892 29567712

[B37] Sicard H. Faubladier M. Noaillac-Depeyre J. Léger-Silvestre I. Gas N. Caizergues-Ferrer M. (1998). The role of the *Schizosaccharomyces pombe* gar2 protein in nucleolar structure and function depends on the concerted action of its highly charged N terminus and its RNA-binding domains. Mol. Biol. Cell. 9, 2011–2023. doi: 10.1091/mbc.9.8.2011 9693363 PMC25453

[B38] Tajrishi M. M. Tuteja R. Tuteja N. (2011). Nucleolin. Commun. Integr. Biol. 4, 267–275. doi: 10.4161/cib.4.3.14884 21980556 PMC3187884

[B39] Talbot N. J. Ebbole D. J. Hamer J. E. (1993). Identification and characterization of *MPG1*, a gene involved in pathogenicity from the rice blast fungus *Magnaporthe grisea* . Plant Cell 5, 1575–1590. doi: 10.1105/tpc.5.11.1575 8312740 PMC160387

[B40] Tuteja R. Tuteja N. (1998). Nucleolin: A multifunctional major nucleolar phosphoprotein. Crit. Rev. Biochem. Mol. Biol. 33, 407–436. doi: 10.1080/10409239891204260 9918513

[B41] Venema J. Tollervey D. (1999). Ribosome synthesis in *Saccharomyces cerevisiae* . Annu. Rev. Genet. 33, 261–311. doi: 10.1146/annurev.genet.33.1.261 10690410

[B42] Wilson R. A. Talbot N. J. (2009). Under pressure: investigating the biology of plant infection by *Magnaporthe oryzae* . Nat. Rev. Microbiol. 7, 185–195. doi: 10.1038/nrmicro2032 19219052

[B43] Xiao S. Caglar E. Maldonado P. Das D. Nadeem Z. Chi A.. (2014). Induced expression of nucleolin phosphorylation-deficient mutant confers dominant-negative effect on cell proliferation. PLoS One 9, e109858. doi: 10.1371/journal.pone.0109858 25313645 PMC4196967

[B44] Xu J. R. Hamer J. E. (1996). MAP kinase and cAMP signaling regulate infection structure formation and pathogenic growth in the rice blast fungus *Magnaporthe grisea* . Genes. Dev. 10, 2696–2706. doi: 10.1101/gad.10.21.2696 8946911

[B45] Xu J. R. Staiger C. J. Hamer J. E. (1998). Inactivation of the mitogen-activated protein kinase Mps1 from the rice blast fungus prevents penetration of host cells but allows activation of plant defense responses. Proc. Natl. Acad. Sci. 95, 12713–12718. doi: 10.1073/pnas.95.21.12713 9770551 PMC22896

[B46] Zhang X. Xiao S. Rameau R. D. Devany E. Nadeem Z. Caglar E.. (2018). Nucleolin phosphorylation regulates PARN deadenylase activity during cellular stress response. RNA Biol. 15, 251–260. doi: 10.1080/15476286.2017.1408764 29168431 PMC5798948

[B47] Zhang H. Xue C. Kong L. Li G. Xu J. R. (2011). A Pmk1-interacting gene is involved in appressorium differentiation and plant infection in *Magnaporthe oryzae* . Eukaryot. Cell 10, 1062–1070. doi: 10.1128/EC.00007-11 21642506 PMC3165448

