# Supplementary material for: The nucleolin MoNsr1 plays pleiotropic roles in the pathogenicity and stress adaptation in the rice blast fungus Magnaporthe oryzae
Source: Front Plant Sci. 2024 Oct 15;15:1482934. doi: 10.3389/fpls.2024.1482934 (PMC11528547; doi:10.3389/fpls.2024.1482934)
Supplement: Supplementary file 1 [file DataSheet1.docx]

*Supplementary information*

**The nucleolin MoNsr1 plays pleiotropic roles in the pathogenicity and stress adaptation in the rice blast fungus *Magnaporthe oryzae***

**Zhen Zhang^1, †^, Mohammad Shafiqul Islam^1,2,†^, Jiuzhi Xia^1^, Xiangyang Feng^1^, Muhammad Noman^1,3,4^, Jing Wang^1^, Zhongna Hao^1^, Haiping Qiu^1^, Rongyao Chai^1^, Yingying Cai^1^, Yanli Wang^1,*^, Jiao-Yu Wang^1*^**

1. Laboratory for Managing Biotic and Chemical Threats to the Quality and Safety of Agro-Products, Key Laboratory of Biotechnology in Plant Protection of Ministry of Agriculture and Rural Affairs, Institute of Plant Protection and Microbiology, Zhejiang Academy of Agricultural Sciences, Hangzhou, 310021, China
2. State Key Laboratory of Rice Biology and Breeding, Ministry of Agriculture Key Laboratory of Molecular Biology of Crop Pathogens and Insects, Key Laboratory of Biology of Crop Pathogens and Insects of Zhejiang Province, Institute of Biotechnology, Zhejiang University, Hangzhou 310058, China
3. Advanced Research Centre, European University of Lefke Lefke, Northern Cyprus, TR-10 Mersin, Turkey
4. Department of Life Sciences, Western Caspian University, Baku, Azerbaijan

* Correspondence: wangjiaoyu78@sina.com

† These authors contributed equally to this work.

Figure S1 Generation of the *MoNsr1* gene deletion mutants and reintroduction mutant. (A) The MoGFD1 was replaced with the HPH cassette by homologous recombination. Primers for PCR screening of gene deletion mutants were labeled on the schematic. (B) Gene deletion mutants were identified by PCR. M for Marker; lane 1,4, and 7 for Guy11; lane 2, 5, and 8 for the gene deletion mutant 1268-5; lane 3, 6, and 9 for the gene deletion mutant 1268-9. Lane 1-3 for PCR product of primers MGG_01268innerF and MGG_01268innerR. Lane 4-6 for PCR product of primers MGG_01268upyzF and HPH-R. Lane 7-9 for PCR product of primers HPH-F and HPH-R. (C) Complementation was identified by PCR with the primers MGG_01268innerF and MGG_01268innerR. M for Marker; lane 1 for Guy11; lane 2 for the gene deletion mutant 1268-5; lane 3-5 for the three complementary strains.

Table S1 Primers used in this study.

| **Primer** | **Sequence (5’-3’)** | **Function** |
| --- | --- | --- |
| MGG_01268upF | gaccatgattacgaattcatcatgtttgcaaatgcgac | Used to Amplify the upstream fragments of the *MoNsr1* gene for the construction of pKO-NSR1. |
| MGG_01268upR | aaggaatagagtagccccaccaagcttttgtagcac |  |
| MGG_01268dnF | gttgacctccaggatccgagggcttctgttcacacct | Used to Amplify the upstream fragments of the *MoNsr1* gene for the construction of pKO-NSR1. |
| MGG_01268dnR | ctgcaggtcgactctagattgagacgaactgaggtgg |  |
| MGG_01268innerF | gccaagtccaagaagatt | Used to verify the presence of the *MoNsr1* gene. |
| MGG_01268innerR | gtcttctcagccttagcc |  |
| MGG_01268upyzF | gtttggtttgctacagcg | Used to verify homologous recombination events in the MoNsr1 gene deletion mutants. |
| HPH-F | tagtggaggtcaacacatcaatgc | Used to Amplify the fragments of HPH in the MoNsr1 gene deletion mutants. |
| HPH-R | catctactctattcctttgccctcgg |  |
| MGG_01268ComF | gaccatgattacgaattcaagctccagtgcgcagagtcg | Used to Amplify the *MoNsr1* gene for complementation. |
| MGG_01268ComR | ctgcaggtcgactctagattgagacgaactgaggtgg |  |
