# Supplementary material for: The nucleolin MoNsr1 plays pleiotropic roles in the pathogenicity and stress adaptation in the rice blast fungus Magnaporthe oryzae
Source: Front Plant Sci. 2024 Oct 15;15:1482934. doi: 10.3389/fpls.2024.1482934 (PMC11528547; doi:10.3389/fpls.2024.1482934)
Supplement: Supplementary file 2 [file Presentation1.pptx]

## Slide 1
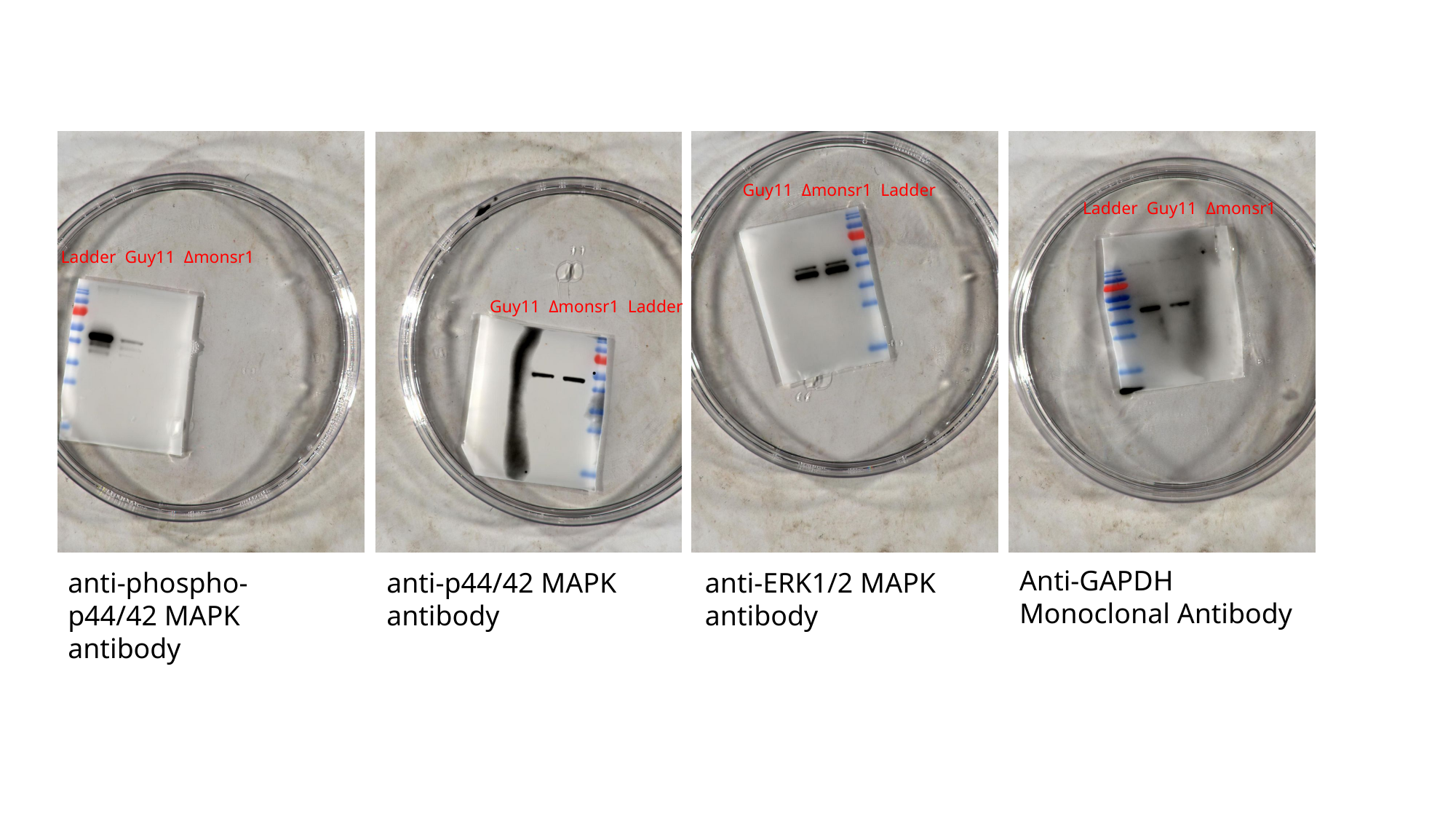

Guy11 Δmonsr1 Ladder
Ladder Guy11 Δmonsr1
Ladder Guy11 Δmonsr1
Guy11 Δmonsr1 Ladder
Anti-GAPDH Monoclonal Antibody
anti-phospho-p44/42 MAPK antibody
anti-p44/42 MAPK antibody
anti-ERK1/2 MAPK antibody
